# A Rare Case of Conjunctival and Scleral Necrosis Following Anterior Sub‐Tenon Triamcinolone Acetonide Injection in a Pediatric Patient

**DOI:** 10.1002/ccr3.72359

**Published:** 2026-03-19

**Authors:** Muhammad Mateen Amir, Minahal Mateen, Bilal Aslam, Shafiq Ur Rahman, Fazeela Bibi, Khalil El Abdi, Said Hamid Sadat

**Affiliations:** ^1^ Alkhidmat Teaching Hospital, Affiliated With University College of Medicine and Dentistry Lahore Pakistan; ^2^ PGR Opthalmology Combined Military Hospital Lahore Pakistan; ^3^ University of Lahore Lahore Pakistan; ^4^ House Officer Opthalmology, Saidu Group of Teaching Hospital Swat Pakistan; ^5^ Jinnah Medical and Dental College Karachi Pakistan; ^6^ Faculty of Medicine and Pharmacy of Rabat Mohammed V University Rabat Morocco; ^7^ Kabul University of Medical Sciences Abu Ali Ibn Sina Kabul Afghanistan

**Keywords:** cataract, child, drug‐related side effects and adverse reactions, injections, intraocular, scleritis, triamcinolone acetonide

## Abstract

The sub‐Tenon route for injecting triamcinolone acetonide is one of the widely practiced surgical techniques for postoperative inflammation control, but complicated conjunctival and scleral necrosis are rare occurrences. A 3‐year‐old boy underwent bilateral cataract surgery and an anterior sub‐tendon injection of triamcinolone (AST) (20 mg). One week later, he presented with conjunctival necrosis at the injection site and thinning of the sclera in both eyes. Necroses being unresponsive to conservative management led to surgical debridement and removal of residual steroid deposits. The conjunctiva healed well without grafting, and the patient remained stable at the 6‐month follow‐up from the ocular point of view. This rare but serious complication of periocular steroid injection in a child demands cautious administration of steroids, prompt diagnosis of necrosis, and timely intervention to limit serious ocular morbidity.

## Key Clinical Message

In children, subconjunctival triamcinolone injections have been reported to cause conjunctival and scleral necrosis. Timely diagnosis and management with prompt withdrawal of steroids, maintenance of ocular surface integrity, and debridement could perhaps ameliorate the eventual dire sequelae. Improvement can promptly be achieved with careful regulation and follow‐up.

## 1 Introduction

Corticosteroids are commonly employed during ophthalmic surgery to manage postoperative inflammation and avoid complications. Sub‐Tenon triamcinolone injection has been a favored approach to managing ocular inflammation as they deliver focal anti‐inflammatory effects with minimal systemic side effects [1]. Although widely practiced, adverse reactions of triamcinolone injection, more specifically scleral necrosis [2], are rare and poorly reported, more so in pediatric cases. Conjunctival necrosis and ischemia due to periocular/intraocular administration of methylprednisolone, triamcinolone acetonide, and betamethasone were already noted in adult patients [3, 4, 5].

Scleral necrosis is a severe complication that can cause progressive thinning of the sclera, tissue loss, and, in some cases, perforation. Scleral necrosis has largely been related to systemic autoimmune diseases, infectious etiologies, and prior ocular surgeries. Yet, localized scleral necrosis after corticosteroid injections has been described, with possible mechanisms being direct cytotoxicity of the drug or its preservatives, localized ischemia secondary to steroid‐induced vasoconstriction, and mechanical trauma from the injection process itself [6]. Drug‐induced scleral necrosis is an underdiagnosed condition, with few reported cases after corticosteroid injections. In adult patients, localized scleral necrosis has been reported following subconjunctival or periocular steroid injections in patients with predisposing conditions like prior surgery or impaired ocular vasculature [7].

Most of the reported cases are adults with pre‐existing predispositions, but cases in pediatric patients continue to be very rare, and this raises fears regarding possible variations in scleral susceptibility and healing patterns in children [8]. Benzyl alcohol experiments have been shown to disrupt the epithelial cell membrane and initiate the process of apoptosis and its resultant delayed healing effects along with inflammation at the wound site [9].

Such an instance has been reported in a child who had undergone the same procedure and developed conjunctival necrosis due to the application of triamcinolone acetonide into her eye through a subconjunctival route for chronic, severe anterior uveitis. This also exists in a child with conjunctival necrosis after performing subconjunctival injection of triamcinolone acetonide for chronic severe anterior uveitis [8].

## 2 Case History

A 3‐year‐old male patient presented to our ophthalmology department with progressive visual deterioration from the last 2 years, and following complete ophthalmic and systemic examination, the patient was diagnosed as a case of congenital bilateral cataract. The laboratory findings for this 3‐year‐old child included complete blood count, blood glucose, serum phosphate, calcium, serum PTH, and ALP were within the normal range for age. Additionally, liver function tests (LFTs), renal function tests (RFTs), and screening for galactosemia were all normal. TORCH screening was also negative. Hence, the case was a bilateral congenital cataract.

He was listed for bilateral cataract surgery under general anesthesia. Surgery was successfully performed with foldable intraocular lens (IOL) implantation. Intraoperatively, an AST of triamcinolone acetonide 20 mg was given for control of postoperative inflammation.

The postoperative course was uncomplicated, with the patient being discharged on topical steroids (dexamethasone, 2‐hourly) and topical antibiotics (tobramycin, 4 hourly).

**FIGURE 1:**
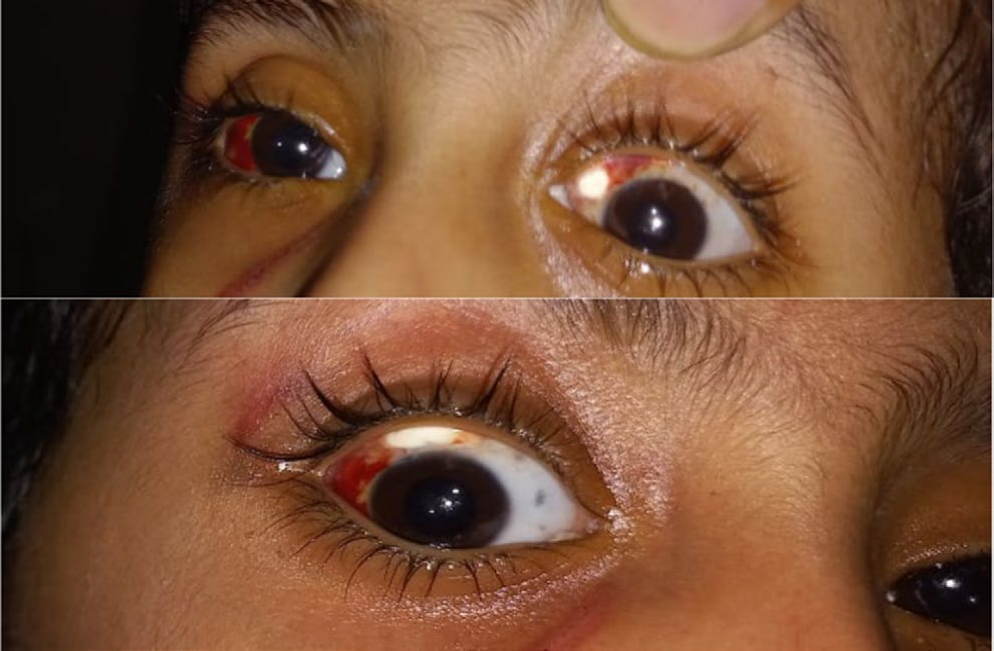
The initial presentation of the patient's eyes, showing regions of conjunctival necrosis and scleral involvement in the supranasal quadrants of both eyes 1.

However, towards the end of the first week after the surgery, the patient developed localized conjunctival necrosis and scleral thinning in both eyes, which were more prominent in the supronasal quadrants (as seen in Figure 1). There was conjunctival ischemia with overlying necrotic tissue and a dull scleral appearance with the presence of early signs of scleral necrosis. The necrotic region was located over the site of AST injection which increased the suspicion of an isolated drug reaction.

Figure 1 demonstrates the initial presentation with regions of conjunctival necrosis and scleral involvement in both supronasal quadrants of the eyes. There was no intraocular inflammation, infection, or hypotony. The patient had no past medical history of autoimmune disease, systemic vasculitis, or past ocular surgery, so the reaction to the triamcinolone injection was the most likely etiology of the necrosis. A conservative strategy was first implemented, involving topical lubrication, antibiotics, and corticosteroids. During the subsequent 3 weeks, the conjunctival necrosis extended with localized scleral exposure but without perforation and anterior chamber reaction or uveal prolapse.

At this time, surgery was contemplated to excise the triamcinolone deposits, which were thought to be causing persistent scleral toxicity. Under local anesthesia, a gentle debridement of necrotic tissue and scraping of residual triamcinolone was performed. The defect unexpectedly was not that big, and the scleral bed was not destroyed, with no urgent requirement for grafting.

**FIGURE 2:**
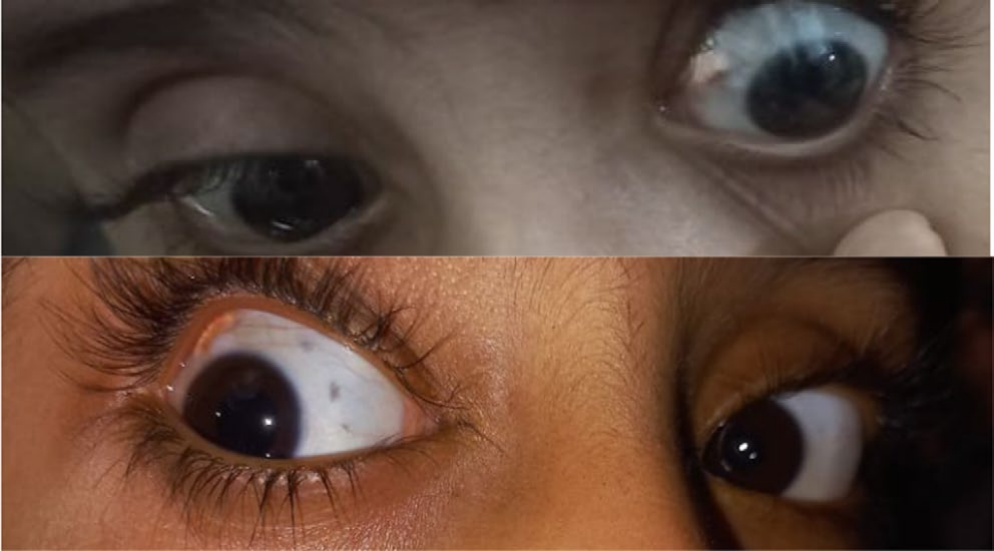
The healed ocular surface after treatment, with regeneration of the conjunctival tissue and resolution of the necrotic zone 2.

Postoperatively, the patient was observed closely, and there was notable improvement within the subsequent few weeks. Figure 2 demonstrates the ocular surface which has healed with regeneration of conjunctival tissue and resolution of the necrotic zone. The integrity of the sclera was maintained, and the vision of the patient remained stable without evidence of recurrence or inflammation.

## 3 Differential Diagnosis

In a 3‐year‐old child, scleritis is rare, and other conditions can mimic its presentation. The differential diagnosis includes but is not limited to the following:

Ocular tuberculosis: Investigations were non‐contributory to aid the diagnosis of ocular tuberculosis since there were no systemic manifestations.

Juvenile idiopathic arthritis (JIA)‐associated uveitis: Can present with red eye and anterior segment inflammation. There was no history of joint disease.

Sarcoidosis: Rare in young children and also systemic and ocular findings were insignificant to support the diagnosis.

Episcleritis: Less painful, sectoral redness, usually self‐limiting. Typical findings of episcleritis were not noticed in this patient and the mentioned findings are supportive of drug‐induced necrosis.

Traumatic scleritis: There was no history of trauma or foreign body.

Wegener's granulomatosis (GPA): Necrotizing scleritis with systemic vasculitis signs was not present and the anti‐neutrophilic cytoplasmic antibody test was negative.

Such conditions must be carefully ruled out before making a diagnosis of conjunctival and scleral necrosis following the said intervention.

## 4 Discussion

Bilateral conjunctival and scleral necrosis after sub‐Tenon triamcinolone injection is a rare but severe complication, especially in children. This 3‐year‐old boy's case underscores the danger of local steroid‐induced tissue necrosis, an experience well described in adult cases but infrequently in children. The mechanisms likely include direct cytotoxicity of triamcinolone and its preservatives, especially benzyl alcohol, which has been associated with ocular surface toxicity and delayed wound healing [1, 9].

Moreover, steroid‐induced vasoconstriction could have potentiated ischemic necrosis in the supronasal quadrants areas with relatively poor vascular density [6, 10]. The patient in this instance had localized conjunctival ischemia and thinning of the sclera at the site of injection, which indicates a local toxic effect of the drug on ocular tissue. Experimental studies have indicated that benzyl alcohol initiates disruption of the epithelial cell membrane and apoptosis, which results in delayed healing and inflammation of the wound [9].

A similar case has been reported in a child with conjunctival necrosis after subconjunctival injection of triamcinolone acetonide for chronic severe anterior uveitis [8]. In a similar case, another case had been reported where there was chronic retention of steroid resulting in progressive thinning of the sclera [6].

In contrast to these cases, our patient had neither systemic risk factors nor previous surgery, pointing toward drug toxicity as the etiology. In addition, though most of the adult cases need grafting, early treatment with drug withdrawal, surgical debridement, and support therapy permitted spontaneous healing without grafting, highlighting the possible conservative management in children with minor necrosis.

This case reminds us that one must remain vigilant while using periocular steroids in children because of the tremendous susceptibility of their tissues and prolonged drug retention in the developing eye.

Given the severity of this complication, strategies to mitigate risk when using periocular steroids in young children are essential. While definitive pediatric dosing guidelines for sub‐Tenon triamcinolone are lacking, the 20 mg dose used in our patient—though reported in older children for other conditions [11, 12]—may warrant re‐evaluation for postoperative prophylaxis in infants and toddlers. Establishing a minimal effective dose should be a research priority. Furthermore, injection technique is a critical variable. The anterior placement of the steroid depot in this case likely contributed to the necrosis, a risk highlighted by complications arising from superficial injections [13]. A more posterior sub‐Tenon injection, a technique widely used to target posterior segment inflammation [14, 15], may be a safer approach to distance the steroid depot from the vulnerable anterior ocular surface.

Exploring therapeutic alternatives is also crucial for enhancing safety. The use of preservative‐free triamcinolone acetonide could reduce direct cytotoxicity, as preservatives like benzyl alcohol are associated with delayed wound healing and ocular toxicity [15, 16]. Other delivery routes, such as intracameral triamcinolone, have been shown to be safe and effective for controlling inflammation after pediatric cataract surgery [17, 18]. For chronic inflammatory conditions, periocular injections may serve as a bridge to systemic immunomodulatory therapies, which remain a cornerstone of long‐term management. This case underscores the urgent need for further research to delineate optimal dosing, safer injection techniques, and less toxic alternatives to control inflammation in the pediatric eye, thereby preventing severe ocular morbidity.

Practitioners must therefore be alert for any early signs of conjunctival ischemia following the injection, which could be instrumental in preventing dangerous scleral thinning and structural complications; Especially in a context where there's a lack of pediatric data for steroid‐induced ocular necrosis, which underlines that further research is needed to determine risk factors, better delineate steroid injection techniques, and investigate alternatives to safer control of postoperative inflammation.

## 5 Conclusion

Sub‐tenon triamcinolone acetonide injection may lead to rare but severe complications like conjunctival and scleral necrosis even in children. Prompt recognition, discontinuation of steroids, ocular surface care, and prompt debridement can result in successful healing without grafting. This case emphasizes the importance of judicious use of steroids, appropriate dosing, and close postoperative observation in children to avoid severe ocular morbidity.

## Author Contributions

**Muhammad Mateen Amir:** conceptualization, data curation, formal analysis, investigation, methodology, project administration. **Minahal Mateen:** conceptualization, data curation, formal analysis, investigation, methodology. **Bilal Aslam:** supervision, validation, writing – original draft, writing – review and editing. **Shafiq Ur Rahman:** investigation, methodology, project administration, visualization, writing – original draft, writing – review and editing. **Fazeela Bibi:** methodology, project administration, visualization, writing – original draft, writing – review and editing. **Khalil El Abdi:** formal analysis, investigation, resources, validation, visualization, writing – original draft, writing – review and editing. **Said Hamid Sadat:** conceptualization, data curation, formal analysis, investigation, methodology.

## Funding

The authors have nothing to report.

## Ethics Statement

The publication of this case report has been authorized by the quality service of our institution, as case reports are exempted from ethical approval in our institute.

## Consent

Written informed consent was obtained from the individual for the publication of any potentially identifiable images or data included in this article.

## Conflicts of Interest

The authors declare no conflicts of interest.

## Data Availability

Due to privacy and ethical restrictions, the data supporting the findings of this study are available from the corresponding author upon reasonable request.
